# Ribose-Map: a bioinformatics toolkit to map ribonucleotides embedded in genomic DNA

**DOI:** 10.1093/nar/gky874

**Published:** 2018-10-01

**Authors:** Alli L Gombolay, Fredrik O Vannberg, Francesca Storici

**Affiliations:** School of Biological Sciences, Georgia Institute of Technology, Atlanta, GA 30332-0230, USA

## Abstract

Recent advances in high-throughput sequencing techniques have made it possible to tag ribonucleoside monophosphates (rNMPs) embedded in genomic DNA for sequencing. rNMP sequencing experiments generate large, complex datasets that require efficient, scalable software that can accurately map embedded rNMPs independently of the particular sequencing technique used. Current computational pipelines designed to map rNMPs embedded in genomic DNA are customized for data generated using only one type of rNMP sequencing technique. To standardize the processing and analysis of rNMP sequencing experiments, we developed Ribose-Map. Through a series of analytical modules, Ribose-Map transforms raw sequencing data into summary datasets and publication-ready visualizations of results, allowing biologists to identify sites of embedded rNMPs, study the nucleotide sequence context of these rNMPs and explore their genome-wide distribution. By accommodating data from any of the available rNMP sequencing techniques, Ribose-Map can increase the reproducibility of rNMP sequencing experiments and enable a head-to-head comparison of these experiments.

## INTRODUCTION

Ribonucleoside monophosphates (rNMPs), the subunits of RNA, are the most common non-canonical nucleotides embedded in genomic DNA. DNA polymerases frequently incorporate rNMPs, instead of deoxyribonucleotides, into genomic DNA during DNA replication and repair (1). In addition, oxidative damage can convert deoxyribonucleotides into rNMPs (2). When left unrepaired, embedded rNMPs can cause several types of genome instability, including increased mutation rates, replication stress and strand breaks (1), and they can also alter DNA structure (3). Although embedded rNMPs are the most frequent distortions found in genomic DNA, there is still much to be learned about the biological mechanisms that regulate the presence of embedded rNMPs in DNA and the effects of embedded rNMPs on genome stability, DNA metabolism and disease.

**Figure 1.**
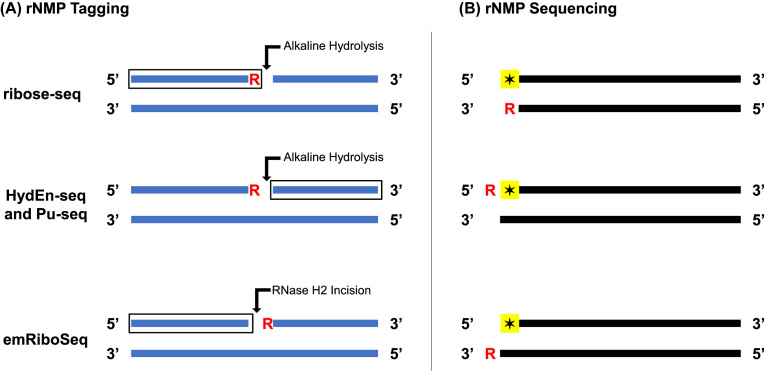
Tagging and sequencing of embedded rNMPs. The positions of embedded rNMPs (R) in double-stranded DNA relative to (**A**) the sites of alkaline hydrolysis or RNase H2 incision (arrow) and the nucleotides captured during library preparation (box) and (**B**) the 5′ tagged nucleotides of the sequencing reads (star) during sequencing for ribose-seq, HydEn-seq, Pu-seq and emRiboSeq. During library preparation, ribose-seq directly captures rNMPs along with the nucleotides upstream from them, HydEn-seq and Pu-seq capture the nucleotides downstream from rNMPs, and emRiboSeq captures the nucleotides upstream from rNMPs. During sequencing, the rNMPs in ribose-seq libraries are the reverse complements of the tagged nucleotides, the rNMPs in Pu-seq and HydEn-seq libraries are one nucleotide upstream from the tagged nucleotides and the rNMPs in emRiboSeq libraries are one nucleotide downstream from the reverse complements of the tagged nucleotides.

To understand the relevance of rNMPs embedded in genomic DNA, it is critical to first know where embedded rNMPs are located in the genome. Recently, high-throughput sequencing techniques were developed to tag and sequence embedded rNMPs. These techniques include (i) embedded ribonucleotide sequencing (emRiboSeq) (4), (ii) hydrolytic end sequencing (HydEn-seq) (5), (iii) ribose-seq (6) and (iv) polymerase usage sequencing (Pu-seq) (7). Each sequencing technique tags embedded rNMPs relative to the 5′ nucleotides of the sequencing reads (referred to as the tagged nucleotides) in one of three ways. emRiboSeq uses the RNase H2 enzyme to make an incision at the 5′ side of embedded rNMPs, thus capturing the nucleotides upstream from rNMPs and placing the rNMPs one nucleotide downstream from the reverse complements of the tagged nucleotides. In contrast, HydEn-seq and Pu-seq use alkali to hydrolyze the DNA backbone at the 3′ side of embedded rNMPs, thus capturing the nucleotides downstream from rNMPs and placing the rNMPs one nucleotide upstream from the tagged nucleotides. In addition to using alkali to cleave at the 3′ side of embedded rNMPs, ribose-seq also takes advantage of *Arabidopsis thaliana* tRNA ligase to directly capture rNMPs along with the nucleotides upstream from them, placing the rNMPs as the reverse complements of the tagged nucleotides. Figure 1 compares the positions of embedded rNMPs in double-stranded DNA during rNMP tagging and sequencing for emRiboSeq, HydEn-seq, ribose-seq and Pu-seq. Although each sequencing technique is unique in how it tags embedded rNMPs, the overall goal remains the same: to map the genome-wide distribution of embedded rNMPs to single-nucleotide resolution. Together, these techniques will help researchers investigate the biological mechanisms that regulate the presence of embedded rNMPs and the effects of embedded rNMPs on genome stability, DNA metabolism and disease.

Achieving the full potential of rNMP sequencing depends upon efficient, scalable software that can accurately map the sites of potentially millions of embedded rNMPs independently of the sequencing technique used. To streamline and standardize the processing and analysis of rNMP sequencing data, we developed Ribose-Map, a comprehensive bioinformatics toolkit that allows biologists to identify sites of embedded rNMPs, study the nucleotide sequence context of these sites and explore their genome-wide distribution. Ribose-Map improves upon current computational pipelines designed to map rNMPs embedded in genomic DNA in three important ways. First, and most significantly, Ribose-Map processes and analyzes data generated from any of the available rNMP sequencing techniques. Second, Ribose-Map requires minimal set-up in the computing environment and depends on only free, open-source software rather than proprietary computer cluster software, allowing it to be run on any Unix/Linux-based computer. Third, Ribose-Map is divided into four analytical modules that together process and analyze rNMP sequencing from start to finish. Ribose-Map is fully documented and regularly maintained by the developers at https://github.com/agombolay/ribose-map.

### Overview of Ribose-Map

The most important task in processing and analyzing rNMP sequencing data is to accurately map the sites of embedded rNMPs in the genome. Once these sites are determined, the door is open to studying many different biological questions regarding the incorporation of rNMPs into genomic DNA. For example, determining if certain types of rNMPs are more frequently incorporated than others and if the presence of embedded rNMPs is influenced by DNA sequence context would help uncover physiological and pathological signatures of rNMP incorporation. In addition, identifying regions in the genome that are enriched with embedded rNMPs would offer clues regarding possible genetic and epigenetic consequences of rNMP incorporation.

**Figure 2.**
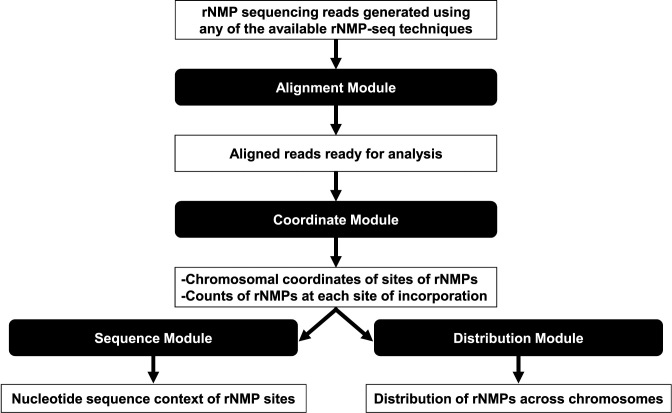
An overview of Ribose-Map. First, the Alignment Module aligns reads to the reference genome of interest (de-duplication based on UMIs and de-multiplexing based on molecular barcodes are also performed if needed). Then, the Coordinate Module uses these aligned reads to calculate the chromosomal coordinates of sites of embedded rNMPs and the counts of rNMPs at each site based on the rNMP sequencing technique used. These coordinates are provided as input into the Sequence and Distribution Modules, which produce data tables, genome browser annotation files and visualizations that can be used to investigate the nucleotide sequence context of the sites of embedded rNMPs and their genome-wide distribution.

To map the sites of embedded rNMPs and enable the biological significance of those sites to be interpreted, Ribose-Map employs a series of four modules to transform rNMP sequencing data generated using any of the available rNMP sequencing techniques into summary datasets and publication-ready visualizations, highlighting the nucleotide sequence context and genome-wide distribution of embedded rNMPs. Figure 2 shows the input/output of the four modules of Ribose-Map (Alignment, Coordinate, Sequence and Distribution) and describes the main functions performed by each module. First, the Alignment Module aligns input rNMP sequencing reads to the reference genome of interest (de-duplication based on unique molecular identifiers (UMIs) and de-multiplexing based on molecular barcodes are also performed if needed). Then, based on the 5′ positions of the aligned reads, the Coordinate Module calculates the chromosomal coordinates of sites of embedded rNMPs and the counts of rNMPs at each site based on the rNMP sequencing technique used. Afterward, the Sequence and Distribution Modules use these coordinates to assess the nucleotide sequence context of the sites of embedded rNMPs and their genome-wide distribution. In addition to mapping rNMPs embedded in genomic DNA, biologists can also use any or all of the modules of Ribose-Map to map other non-canonical nucleotides to single-nucleotide resolution.

## MATERIALS AND METHODS

Since rNMP sequencing and mapping is a new and rapidly growing area of research and consensus regarding how to process and analyze rNMP sequencing data is currently lacking, we designed Ribose-Map to serve as the first all-inclusive, ready-to-use, standardized bioinformatics toolkit to efficiently and accurately map sites of rNMPs embedded in genomic DNA that were tagged and sequenced using any of the available rNMP sequencing techniques. Through a series of four modules (Alignment, Coordinate, Sequence and Distribution Modules), Ribose-Map transforms rNMP sequencing data into publication-ready visualizations of results, highlighting the nucleotide sequence context and distribution of sites of rNMPs embedded in genomic DNA. All of the modules of Ribose-Map are either novel in function or at least provide greater ease of use than current rNMP mapping software, allowing both experienced and novice bioinformatics users to easily process and analyze any type of rNMP sequencing data. In the following section, we describe the four modules of Ribose-Map and highlight key features that should be considered when processing and analyzing rNMP sequencing data.

To download and install Ribose-Map and its software dependencies, the Ribose-Map GitHub repository should be cloned from https://github.com/agombolay/ribose-map, and the Ribose-Map conda software environment should be created via MiniConda and BioConda (BioRxiv: https://doi.org/10.1101/207092) on Linux or Mac OSX. Instructions and data files needed to create the conda software environment are provided in the Ribose-Map GitHub repository (see Data Availability).

### Read alignment with Alignment Module

#### Command-line usage for Alignment Module: ribose-map alignment config

Analysis of rNMP sequencing data begins by aligning reads to the reference genome of interest. Using Bowtie 2 (8), the Alignment Module of Ribose-Map aligns single- or paired-end sequencing reads to the reference genome. The reads should be high-quality sequences devoid of laboratory contaminants, sequencing adapters and low-quality base calls. If necessary, the reads should be processed with cutadapt (9) or similar data cleaning software to remove any artifacts before alignment.

In addition to aligning the reads, the Alignment Module also de-multiplexes the reads based on molecular barcodes and de-duplicates the aligned reads based on UMIs if needed. When preparing rNMP sequencing libraries, molecular barcodes can be used to separate reads containing embedded rNMPs from background noise, while UMIs can be used to remove polymerase chain reaction (PCR) artifacts from the data. Since PCR amplification is known to be biased, aligned reads generated from rNMP sequencing libraries that were created using PCR should be de-duplicated prior to downstream analyses to prevent incorrect and/or misleading results. For example, if read X is amplified disproportionately more than read Y, then rNMPs would appear to be more frequently incorporated in the region to which read X aligns than the region to which read Y aligns. According to Smith *et al.*, disproportionate representation of reads due to PCR amplification is more pronounced when greater numbers of PCR cycles are used and when the alignment coordinates are concentrated in only certain locations (10). Since three of the rNMP-seq techniques use PCR (and at least one technique uses ≥15 cycles) and the alignment coordinates of embedded rNMPs are often concentrated in certain locations, not de-duplicating the data based on UMIs could significantly alter the biological findings of rNMP-seq experiments. Therefore, rNMP-seq data should be de-duplicated based on UMIs before conducting downstream analyses. To account for biased PCR amplification, UMIs, which are random oligonucleotide tags, can be attached to the 5′ ends of reads, allowing identical reads originating from distinct molecules to be distinguished from those originating from PCR amplification of the same molecule and then de-duplicated accordingly. In particular, identical reads originating from distinct molecules would align to the same 5′ position in the genome and have different UMIs and thus should be retained, while those originating from PCR amplification of the same molecule would align to the same 5′ position in the genome but have the same UMI and thus should be de-duplicated.

If the reads were tagged with molecular barcodes, the Alignment Module retains only those reads containing the relevant barcodes and then removes those barcodes from the reads using seqtk (https://github.com/lh3/seqtk) in preparation for alignment. If the reads were tagged with UMIs during library preparation, the Alignment Module uses UMI-tools (10) to extract the UMIs from the reads and append them to the read names prior to alignment to the reference genome. After alignment, the Alignment Module uses UMI-tools to group reads that are likely PCR duplicates based on both their aligned positions and UMIs and then de-duplicate those reads to yield one read per group.

### Locating sites of embedded rNMPs with the Coordinate Module

#### Command-line usage for Coordinate Module: ribose-map coordinate config

Once the reads are processed and aligned to the reference genome of interest, the Coordinate Module determines the chromosomal coordinates (i.e. chromosome name, start/end position and DNA strand) of sites of embedded rNMPs and the counts of rNMPs at each site for any of the available rNMP sequencing techniques. The chromosomal coordinates are calculated relative to the 5′ tagged nucleotides of the reads (read 1). Depending on the length of the DNA fragments and the read length used, next-generation sequencing instruments may not sequence the entire length of the fragments starting from their 5′ side and ending at their 3′ side. Thus, the embedded rNMPs must be tagged relative to the 5′ nucleotides of the reads rather than the 3′ nucleotides to ensure their exact locations can be determined.

To determine the chromosomal coordinates of sites of embedded rNMPs, the Coordinate Module first converts the BAM file containing the alignment data into a BED file containing the zero-based start/end positions of the aligned reads using BEDTools (11). Then, the Coordinate Module uses custom code to calculate the single-nucleotide chromosomal coordinates of sites of embedded rNMPs relative to the 5′ positions of the aligned reads for data generated using any of the available rNMP sequencing techniques (Table 1). In addition to calculating the chromosomal coordinates of rNMPs, the Coordinate Module also screens the coordinates for biological relevance. Although likely rare, contamination or inefficiencies in emRiboSeq, HydEn-seq and Pu-seq could generate reads that align to the 5′-most ends of a given chromosome. Since sites of embedded rNMPs captured by emRiboSeq, HydEn-seq and Pu-seq are located either upstream from the tagged nucleotides or downstream from the reverse complements of the tagged nucleotides, reads that align to the 5′-most positions of the chromosome for any of these sequencing techniques would cause rNMP mapping software to calculate coordinates that are located beyond the ends of the chromosome (and thus biologically meaningless) unless the software also screens the coordinates it calculates for biological relevance prior to saving them to the output BED file. If biologically meaningless coordinates (e.g. −1) were input into the Sequence and Distribution Modules of Ribose-Map or other downstream analytical tools, these programs would produce errors or even error out. Therefore, rNMP mapping software should screen the coordinates of rNMPs prior to saving them to the output BED file as Ribose-Map does to prevent errors in downstream analyses.

**Table 1.** Genomic arithmetic used in the Coordinate Module to calculate chromosomal coordinates of embedded rNMPs

	rNMPs on forward strand		rNMPs on reverse strand	
Technique	Start position	End position	Start position	End position
ribose-seq	end position (–) – 1	end position (–)	start position (+)	start position (+) + 1
HydEn-seq and Pu-seq	start position (+) – 1	start position (+)	end position (–)	end position (–) + 1
emRiboSeq	end position (–)	end position (–) + 1	start position (+) – 1	start position (+)

Chromosomal coordinates of embedded rNMPs are calculated based on the start/end positions of the reads (read 1) aligned to the reference genome. (+) = reads aligned to the forward strand of the reference genome and (–) = reads aligned to the reverse strand of the reference genome.

When calculating the chromosomal coordinates of the embedded rNMPs, three factors must be considered: (i) the rNMP sequencing technique used, (ii) the chromosomal coordinate system and (iii) the strand of DNA to which the read aligned. First, since each rNMP sequencing technique tags embedded rNMPs relative to the 5′ tagged nucleotides differently, the genomic arithmetic used to calculate the chromosomal coordinates of the embedded rNMPs must be specific to the technique used. Second, two related but distinct coordinate systems are used in bioinformatics: zero-based and one-based. In the zero-based coordinate system, counting starts at 0, while in the one-based coordinate system, counting starts at 1. Thus, when calculating the coordinates of the embedded rNMPs, it is important to remember BEDTools uses a zero-based starting position and one-based ending position to label the start/end positions of reads (e.g. the start position of the first nucleotide on a given chromosome would be 0 and the end position would be 1). Third, although the 5′ ends of the forward and reverse strands of DNA are located at opposite ends from one another, the coordinate system begins counting at the 5′ end of the forward strand and the 3′ end of the reverse strand. Therefore, when calculating the coordinates of the embedded rNMPs, it is important to keep in mind the 5′ base of reads that align to the forward strand will correspond to the start position of the reads, while the 5′ base of reads that align to the reverse strand will correspond to the end position of the reads.

### Studying embedded rNMPs with the Sequence and Distribution Modules

#### Command-line usage for Sequence Module: ribose-map sequence config

##### Command-line usage for Distribution Module: ribose-map distribution config

After the chromosomal coordinates of embedded rNMPs are determined, the Sequence and Distribution Modules can be used to explore three important questions: (i) Are there biases in the types of rNMPs (rAMP, rCMP, rGMP or rUMP) embedded in genomic DNA? (ii) Is the presence of embedded rNMPs influenced by DNA sequence context? (iii) Are there regions in the genome that are enriched with embedded rNMPs?

To identify possible biases in the types of rNMPs embedded in DNA and to understand the surrounding DNA sequence context, the Sequence Module calculates and plots the frequencies of the nucleotides at the sites of embedded rNMPs and up/downstream from those sites. The nucleotide frequencies are normalized to the frequencies of the reference genome sequence to allow the user to readily identify if certain types of rNMPs are more frequently incorporated into genomic DNA than others and if the presence of embedded rNMPs is influenced by DNA sequence context. Since the nucleotide sequence context of embedded rNMPs in the nucleus and mitochondria might vary, the plots are separated according to organelle.

To locate regions in the genome that are enriched with embedded rNMPs, the Distribution Module calculates and plots the per-nucleotide coverage of embedded rNMPs across each chromosome. The coverage is calculated by normalizing read counts to read counts per hundred to allow the user to readily compare sequencing libraries of different read depth. In addition, the Distribution Module creates BedGraph files that can be directly uploaded to a genome browser, such as the University of California, Santa Cruz Genome Browser (http://genome.ucsc.edu/), as custom annotation tracks.

## RESULTS AND DISCUSSION

We performed both qualitative and quantitative comparisons of Ribose-Map and the current computational pipelines available to analyze rNMP sequencing data. First, we performed a qualitative comparison of Ribose-Map, emRiboSeqProcessor (4) (created for emRiboSeq data), Modmap (6) (created for ribose-seq data) and the Pu-seq pipeline (7) (created for Pu-seq data). The HydEn-seq pipeline (5) (created for HydEn-seq data) was not included in this comparison because its source code is not publicly available. Next, we performed a quantitative comparison of the only two computational pipelines that produce directly comparable output, Ribose-Map and emRiboSeqProcessor. Evaluations were completed on a MacBook Pro with a 3.1 GHz Intel Core i7. The quantitative comparison assumes a computer with at least two cores to allow for multithreading of processes. Since emRiboSeqProcessor uses two cores when multithreading, two cores were also used for Ribose-Map to allow the performances of these two pipelines to be compared independently of the number of cores used. Parameters used to run Ribose-Map for these analyses are provided in the configuration files that are included in the Supplementary Data at NAR online. The sacCer3 version of the *Saccharomyces cerevisiae* genome was used to create the synthetic datasets and served as the reference genome for the quantitative comparison.

### Qualitative comparison

To evaluate the flexibility, functionality, accessibility and ease-of-use of Ribose-Map compared to emRiboSeqProcessor, Modmap and the Pu-seq pipeline, we examined the source code of each computational pipeline for features that would enhance the pipeline's ability to process and analyze rNMP sequencing data. In particular, we applied the following four evaluation criteria: (1a) analyzes rNMP sequencing data generated using any of the available rNMP sequencing techniques, (1b) analyzes rNMP sequencing data derived from any organism that has a sequenced reference genome, (2a) outputs a BED file containing chromosomal coordinates of rNMP sites that can be used for downstream analyses, (2b) normalizes per-nucleotide counts of embedded rNMPs to account for differences in read depth among sequencing libraries, (3) depends on only free, open-source software, and (4) allows the user to create a ready-to-use software environment in which all software dependencies have been installed. Of the four evaluation criteria used to assess flexibility, functionality, accessibility and ease-of-use (criteria 1, 2, 3 and 4), only Ribose-Map met all four of the criteria.

### Criteria 1: flexibility

Ribose-Map is the only rNMP mapping software that can analyze rNMP sequencing data generated using any of the rNMP sequencing techniques. emRiboSeqProcessor, Modmap and the Pu-seq pipeline are limited to analyzing data generated using emRiboSeq, ribose-seq and Pu-seq, respectively. In addition, Ribose-Map, along with the Pu-seq pipeline, is immediately compatible with rNMP sequencing data generated from any organism that has a sequenced reference genome. Although Modmap and emRiboSeqProcessor could be modified to analyze data derived from organisms other than *S. cerevisiae*, these two pipelines are not flexible enough in their current state to analyze data derived from any organism.

### Criteria 2: functionality

Ribose-Map, along with emRiboSeqProcessor, outputs a BED file containing the chromosomal coordinates of rNMP sites that can be used for downstream analyses, such as for the Sequence and Distribution Modules of Ribose-Map. In contrast, Modmap outputs BedGraph files containing counts of rNMPs per position and the Pu-seq pipeline outputs CSV files containing counts of rNMPs per window. In addition, Ribose-Map, along with emRiboSeqProcessor and the Pu-seq pipeline, normalizes the per-nucleotide counts of embedded rNMPs to account for differences in read depth among sequencing libraries. In contrast, Modmap does not normalize to account for read depth, hindering the comparison of sequencing libraries of different read depth.

### Criteria 3: accessibility

Ribose-Map, along with emRiboSeqProcessor and the Pu-seq pipeline, depends on only free, open-source software. In contrast, Modmap depends on proprietary computer cluster software that is available to only a limited number of researchers.

### Criteria 4: ease-of-use

Ribose-Map is the only rNMP mapping software that allows the user to quickly and easily create a conda software environment in which all software dependencies are installed in one step. The other rNMP mapping software requires the user to install all software dependencies one by one, which is often a tedious and time-consuming process.

Table 2 shows the results of evaluating the flexibility, functionality, accessibility and ease-of-use of Ribose-Map and the current rNMP mapping software. Based on these results, Ribose-Map can more easily and accurately process and analyze a wider variety of rNMP sequencing data than emRiboSeqProcessor, Pu-seq and Modmap individually or combined.

**Table 2.** Qualitative comparison of Ribose-Map and the current rNMP mapping software

	Ribose-Map	Modmap	emRiboSeq Processor	Pu-seq Pipeline
**1. Flexibility**				
(a) Processes and analyzes data generated using any high-throughput rNMP sequencing technique	✓	✗	✗	✗
(b) Processes data derived from any organism	✓	✗	✗	✓
				
**2. Functionality**				
(a) Outputs BED file containing sites of rNMPs	✓	✗	✓	✗
(b) Normalizes counts of rNMPs for read depth	✓	✗	✓	✓
				
**3. Accessibility**				
- Depends on only free, open-source software	✓	✗	✓	✓
				
**4. Ease-of-use**				
- Allows user to create a software environment	✓	✗	✗	✗

### Quantitative comparison

Since Ribose-Map and emRiboSeqProcessor are the only two computational pipelines that produce comparable output, we evaluated the performance of only these two computational pipelines to allow for the most direct comparison. Using both synthetic and real datasets, we ran the Alignment and Coordinate Modules of Ribose-Map and the emRiboSeqProcessor script to produce a BED file containing the chromosomal coordinates of rNMP sites and tab-delimited files of raw and normalized rNMP counts. To evaluate the ability of Ribose-Map to accurately and efficiently produce these output data files compared to emRiboSeqProcessor, we applied the following three evaluation criteria: (i) accuracy, (ii) execution time and (iii) maximum resident set size.

### Evaluation on synthetic datasets

To test the ability of Ribose-Map and emRiboSeqProcessor to accurately calculate the chromosomal coordinates of rNMP sites and then screen the coordinates for biological relevance prior to saving them to the output BED file, we created two synthetic emRiboSeq datasets, Test Datasets 1 and 2 (see Data Availability). Both datasets consist of 10 unique DNA sequences derived from the *S. cerevisiae* genome. Test Dataset 1 consists of 10 reads that originate from regions other than the 5′-most ends of the chromosome, while Test Dataset 2 contains 8 reads that originate from regions other than the 5′-most ends of the chromosome and 2 reads that align to the 5′-most ends of the chromosome. Test Dataset 2 was created in contrast to Test Dataset 1 to test the degree to which Ribose-Map and emRiboSeqProcessor could not only accurately calculate the chromosomal coordinates of rNMP sites, but also screen the coordinates for biological relevance prior to saving them to the output BED file.

Of the two synthetic datasets that were used to test the accuracy of Ribose-Map and emRiboSeqProcessor, Ribose-Map accurately calculated all of the chromosomal coordinates of rNMP sites in both Test Datasets 1 and 2 and also screened the coordinates for biological relevance. In contrast, emRiboSeqProcessor accurately calculated all the chromosomal coordinates of rNMP sites in both Test Datasets 1 and 2, but did not screen the coordinates for biological relevance, resulting in two biologically meaningless coordinates in the output BED file for Test Dataset 2 (Table 3). These results demonstrate that Ribose-Map, but not emRiboSeqProcessor, is able to accurately calculate the chromosomal coordinates in both possible scenarios that could arise when processing and analyzing real rNMP sequencing data.

**Table 3.** Chromosomal coordinates of rNMPs sites calculated by emRiboSeqProcessor that are located beyond the ends of the chromosomes

Chromosome	Start position	End position	Strand
1. Chromosome I	–1	0	–
2. Chromosome II	813184	813185	+

Coordinates were calculated based on (1.) one read that aligned to the 5′-most end of the forward strand of chromosome I (zero-based start/end positions should be 0/1) and (2.) another read that aligned to the 5′-most end of the reverse strand of chromosome II (zero-based start/end positions should be 813183/813184) of the *Saccharomyces cerevisiae* genome. To prevent errors in downstream analyses, emRiboSeqProcessor should have screened the calculated coordinates for biological relevance prior to saving them to the output BED file.

### Evaluation on emRiboSeq datasets

Next, we evaluated the performances of Ribose-Map and emRiboSeqProcessor using two previously published emRiboSeq datasets, SRR1734964 and SRR1734965. Both datasets contain sequencing reads derived from the genomic DNA of RNase H2-defective *S. cerevisiae* cells and sequenced using Ion Torrent PGM. The SRR1734964 dataset consists of 5,104,023 single-end reads, and the SRR1734965 dataset consists of 6,046,964 single-end reads. The reads do not contain UMIs or molecular barcodes (since emRiboSeq does not use PCR, UMIs are not needed). To quantify the performance of Ribose-Map compared to emRiboSeqProcessor, both pipelines were run five times and the average accuracy, execution time and maximum resident set size were calculated. Using the BED files containing the chromosomal coordinates of rNMP sites that were produced by emRiboSeqProcessor as benchmarks, the Alignment and Coordinate Modules of Ribose-Map identified 100% of the same coordinates for both datasets in an average of 10.85% less time for dataset SRR1734964 and 12.77% less time for dataset SRR1734965 and using similar memory compared to emRiboSeqProcessor. Table 4 shows the results of evaluating the performances of Ribose-Map and emRiboSeqProcessor using the two emRiboSeq datasets. These results demonstrate that Ribose-Map can be used as a more time-efficient alternative to emRiboSeqProcessor to accurately calculate the chromosomal coordinates of sites of embedded rNMPs.

**Table 4.** Quantitative comparison of the performances of Ribose-Map and emRiboSeqProcessor using two previously published emRiboSeq datasets

	Ribose-Map	emRiboSeqProcessor
**emRiboSeq Dataset SRR1734964**		
**(i)** Accuracy	100%	—
**(ii)** Program execution time	8.18 min (0.05)	9.17 min (0.06)
**(iii)** Maximum resident set size	47.55 MB (0.03)	47.62 MB (0.09)
		
**emRiboSeq Dataset SRR1734965**		
**(i)** Accuracy	100%	—
**(ii)** Program execution time	8.77 min (0.07)	10.05 min (0.14)
**(iii)** Maximum resident set size	46.84 MB (0.16)	46.80 MB (0.05)

Performance measurements represent the average (standard deviation) of five replicates.

**Figure 3.**
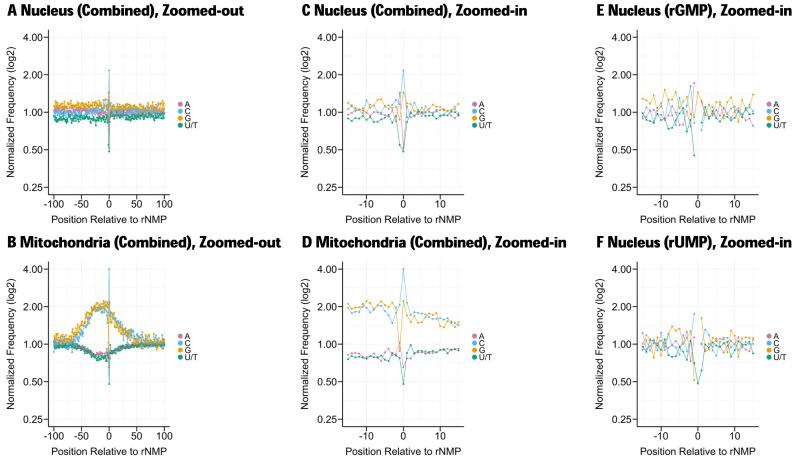
Nucleotide sequence context of rNMPs embedded in the nuclear and mitochondrial DNA of a RNase H2-defective strain of *Saccharomyces cerevisiae* obtained using the Sequence Module of Ribose-Map. Embedded rNMPs were tagged and sequenced using ribose-seq. The nucleotide frequencies are normalized to those of the reference genome. The nucleotide frequencies are shown relative to the sites of embedded rNMPs (position 0 = rNMP site). (**A** and **B**) Zoomed-out (100 bases up/downstream from rNMP sites) for all rNMPs combined, (**C** and **D**) zoomed-in (15 bases up/downstream from rNMP sites) for all rNMPs combined, (**E**) zoomed-in for rGMP and (**F**) zoomed-in for rUMP.

Once the chromosomal coordinates of the sites of embedded rNMPs have been obtained using the Alignment and Coordinate Modules, the user can then use the Sequence Module of Ribose-Map to explore the nucleotide sequence context at the sites of embedded rNMPs and surrounding those sites in both the nucleus and mitochondria to determine if certain types of rNMPs are more frequently incorporated than others in either organelle and if the presence of embedded rNMPs is influenced by DNA sequence context (Figure 3A–D). For example, in a previously published ribose-seq dataset (SRR1575897), rCMP is more frequently incorporated in both the nuclear and mitochondrial DNA. In contrast to the nucleus, rNMPs are markedly abundant in GC-rich regions of the mitochondria. In addition to determining the nucleotide sequence context without respect to the type of rNMP (rAMP, rCMP, rGMP and rUMP combined) as shown in Figure 3A–D, the Sequence Module also determines the nucleotide sequence context with respect to the type of rNMP (rAMP, rCMP, rGMP and rUMP individually), allowing the user to determine whether the DNA sequence context surrounding sites of embedded rNMPs depends on the identity of the embedded rNMP (Figure 3E and F). For example, rGMP (Figure 3E) is most frequently preceded by deoxyribonucleotide A in the nucleus, while rUMP (Figure 3F) is most frequently preceded by deoxyribonucleotide C, suggesting a possible signature of rNMP incorporation. Comparing the sequence context of embedded rNMPs in different species and under different conditions might uncover physiological and pathological signatures of embedded rNMPs.

**Figure 4.**
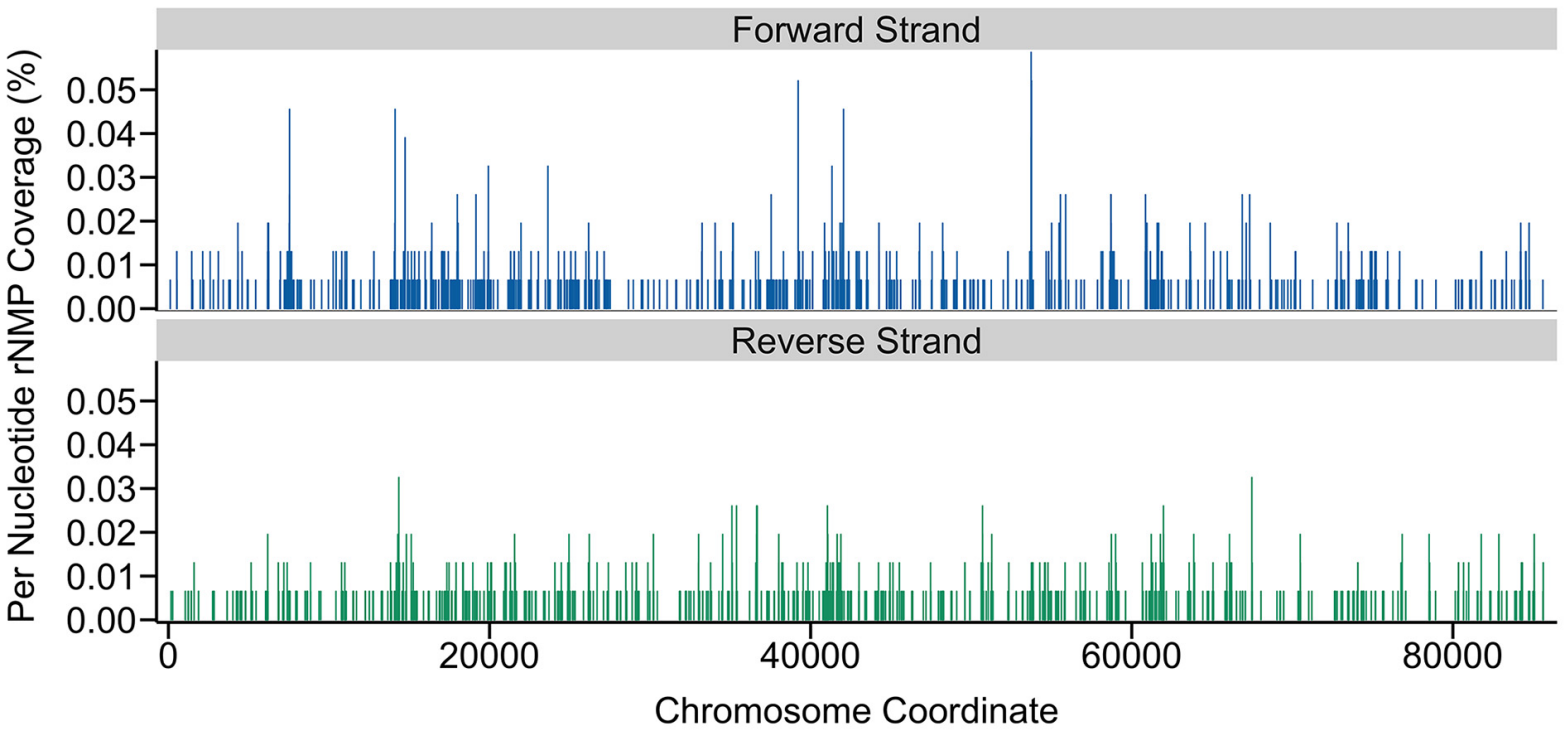
Distribution of rNMPs embedded in the mitochondrial DNA of a RNase H2-defective strain of *Saccharomyces cerevisiae* obtained using the Distribution Module of Ribose-Map. Embedded rNMPs were tagged and sequenced using ribose-seq. The per-nucleotide coverage of rNMPs is normalized to reads per hundred (%).

Using the Distribution Module of Ribose-Map, the user can explore regions that are enriched with embedded rNMPs. First, the user can examine the plots displaying the normalized per-nucleotide coverage of rNMPs to quickly identify chromosome-specific regions that are enriched with embedded rNMPs. For example, in the SRR1575897 ribose-seq dataset, while embedded rNMPs are present throughout the mitochondria, several regions appear to be highly susceptible to embedded rNMPs (Figure 4), all of which warrant further investigation. Next, the user can upload the BedGraph files of rNMP counts to a genome browser and cross-reference these regions with any type of annotation data of interest (e.g. origins of replication). Cross-referencing these regions with annotation data could help reveal the biological mechanisms that regulate the presence of rNMPs in genomic DNA and their effects on genome stability, DNA metabolism and disease.

## CONCLUSION

The recent development of emRiboSeq, HydEn-seq, ribose-seq and Pu-seq has necessitated the development of a standardized, user-friendly computational pipeline that can efficiently and accurately process and analyze any type of rNMP sequencing data. In this work, we used the novel bioinformatics toolkit, Ribose-Map, to demonstrate how to efficiently and accurately transform any type of rNMP sequencing data into summary datasets and publication-ready visualizations of results. When tested on both synthetic and real datasets, Ribose-Map accurately calculated the chromosomal coordinates of rNMP sites in an average of 10.85% less time for dataset SRR1734964 and 12.77% less time for dataset SRR1734965 than emRiboSeqProcessor while maximizing flexibility, functionality, accessibility and ease-of-use, thereby advancing the field of rNMP mapping.

## DATA AVAILABILITY

Ribose-Map is available for download at https://github.com/agombolay/ribose-map. The synthetic datasets used for this study are available as Supplementary Data at NAR online. The emRiboSeq datasets used for this study are available in NCBI’s SRA at the following accession codes: SRR1734964 and SRR1734965, and the ribose-seq dataset used for this study is available in NCBI’s SRA at the following accession code: SRR1575897.

## Supplementary Material

Supplementary Data
